# Two-year experience of the implementation of a psychiatric home hospitalization care service for acute mental illness

**DOI:** 10.1192/j.eurpsy.2023.408

**Published:** 2023-07-19

**Authors:** A. Guàrdia, L. Marin, A. González-Rodríguez, V. Bañon, E. Izquierdo, L. Lafuente, X. Martinez-Bio, D. Llors, M. Natividad, L. Ros, J. A. Monreal

**Affiliations:** ^1^Psychiatry; ^2^Psychiatria, Hospital Universitari Mutua de Terrassa, Terrassa, Spain

## Abstract

**Introduction:**

Psychiatric home hospitalisation is a service aiming to support people with mental illnesses in their acute stage at their own home. This care model has been recently implemented in our territory with the main objective of avoiding hospital admissions.

**Objectives:**

Our goal is to describe a cohort of patients followed up over 2-years in the context of a pilot mental health program within a community-based model (Mutua Terrassa University Hospital).

**Methods:**

We conducted a prospective longitudinal study including 125 patients attended from 01/11/2020 to 09/11/2022 in our reference area of 250,000 inhabitants. The team was formed by 1 psychiatrist and 1 mental health nurse. DSM-5 diagnoses, socio-demographic variables, mean stay and care trajectories were collected.

**Results:**

One-hundred twenty-five patients were attended (women: 70). Mean age at consultation: 38.3 years-old. Mean stay: 24 days. The most frequent diagnoses: non-affective psychotic disorders (58%), affective disorders (30%), followed by anxiety and personality disorders. Referrals from Community Mental Health Outpatient Services (CMHS) (72%), Acute Inpatient Unit (25%), and Psychiatric Emergency Service (3%). Referrals after discharge: CMHS (83%), Adult Acute Inpatient Unit (13%), others (4%). Individualized mental health plans were carried out in all cases, in coordination with community mental health services. Follow-up adherence after discharge was about 95%. Patients with first-episode of psychosis showed the highest degree of satisfaction (N=46).

**Conclusions:**

Patients with emerging psychosis were the profile of users who showed the highest benefit of our service. Women showed higher adherence, and loss to follow-up was lower than we expected.

**Disclosure of Interest:**

None Declared

